# Analysis of Bacterial Biofilm Formation and *MUC5AC* and *MUC5B* Expression in Chronic Rhinosinusitis Patients

**DOI:** 10.3390/jcm12051808

**Published:** 2023-02-23

**Authors:** Georgi Popov, Radoslav Aleksandrov, Veronika Petkova, Radka Kaneva, Raina Gergova, Todor Kundurzhiev, Diana Popova

**Affiliations:** 1 Department of Ear, Nose and Throat Diseases, Medical Faculty, Medical University of Sofia, University Hospital “Tsaritsa Yoanna—ISUL”, Byalo More Str. 8, 1527 Sofia, Bulgaria; 2 Laboratory of Genomic Stability, Institute of Molecular Biology, Bulgarian Academy of Sciences, Georgi Bonchev Bl. 21, 1113 Sofia, Bulgaria; 3 Molecular Medicine Center, Department of Medical Chemistry and Biochemistry, Medical Faculty, Medical University of Sofia, Zdrave Str. 2, 1431 Sofia, Bulgaria; 4 Department of Medical Microbiology, Medical Faculty, Medical University of Sofia, Zdrave Str. 2, 1431 Sofia, Bulgaria; 5 Department of Occupational Medicine, Faculty of Public Health, Medical University of Sofia, University Hospital “Tsaritsa Yoanna—ISUL”, Byalo More Str. 8, 1527 Sofia, Bulgaria

**Keywords:** chronic rhinosinusitis, spinning disk confocal microscopy, qRT-PCR, bacterial biofilm infection, *MUC5AC*, *MUC5B*, endoscopic sinus surgery

## Abstract

Chronic rhinosinusitis (CRS) is a condition affecting as much as 16% of the adult population in developed countries with many factors attributed to its development, including the more recently proposed role of bacterial biofilm infections. Plenty of research has been conducted on biofilms in CRS and the causes behind the development of such an infection in the nasal cavity and sinuses. One such probable cause is the production of mucin glycoproteins by the mucosa of the nasal cavity. To investigate the possible link between biofilm formation and mucin expression levels and their relationship with CRS etiology, we examined samples from 85 patients by means of spinning disk confocal microscopy (SDCM) to establish their biofilm status and quantitative reverse transcription polymerase chain reaction (qRT-PCR) to determine *MUC5AC* and *MUC5B* expression levels. We observed a significantly higher prevalence of bacterial biofilms in the CRS patient group compared to the control group. In addition, we detected higher expression levels of *MUC5B* but not *MUC5AC* in the CRS group, which suggested a possible role for MUC5B in CRS development. Finally, we found no direct relationship between biofilm presence and mucin expression levels, thereby showing a multifaceted connection between these two major factors implicated in CRS etiology.

## 1. Introduction

Chronic rhinosinusitis (CRS) is a condition of chronic inflammation of the nasal cavity and paranasal sinuses that affects as much as 16% of the adult population in the United States [1] and 10.9% in Europe overall, with the percentage in different European countries varying widely and reaching as high as 27.1% [2,3]. CRS causes a significant reduction in the quality of life of affected individuals [4] and imposes serious costs on healthcare systems and society as a whole; the direct costs in the USA alone are estimated to be between USD 10 and USD 13 billion per year and the indirect ones at more than USD 20 billion annually [5].

CRS is diagnosed when two or more of the following symptoms are present for 12 or more weeks in a patient: mucopurulent drainage, nasal obstruction (congestion), facial pain, pressure or fullness, or decreased sense of smell and inflammation documented by two or more of the following findings: purulent mucus or edema in the middle nasal meatus or anterior ethmoid region, polyps in the nasal cavity, and radiographic findings of inflammation [6].

The treatment of CRS is by medication [7]—classically including antibiotics, nasal or systemic steroids, saline, etc.—or by surgery [8], and ideally it should target a major underlying cause if such can be identified in individual patients. Novel therapies including immune modulation and topical agents featuring antibacterial and antibiofilm properties are constantly being pursued [9].

Historically, one of the obstacles in the study of the effects of bacterial biofilm (BBF) formation on chronic rhinosinusitis (CRS) and other pathologies has been and still is the need for specific methods in order to observe the three-dimensional biofilm structure. Usually, scanning electron microscopy (SEM) or confocal scanning laser microscopy (CSLM) complemented by fluorescence in situ hybridization (FISH) or by the use of non-species-specific dyes [10] have been used to elucidate biofilm formation and structure. Standard swab and culturing has often failed to show the presence of bacteria even when they have formed a BBF in different types of pathology [11,12,13,14]; therefore, not only is the formation of a biofilm structure frequently missed, but the bacterial “contribution” to the examined pathology also is overlooked. Due to its sensitivity and specificity, the polymerase chain reaction (PCR) is sometimes used to confirm the presence of bacteria in otherwise negative cultures [13,15], but it cannot prove a biofilm structure on its own.

Over the last decades, multiple studies that used the aforementioned methods yielded substantial evidence that BBFs play a role in the pathogenesis of chronic rhinosinusitis (CRS) [10,16,17,18]. Patients discovered to have a biofilm infection exhibited worse clinical symptom scores [19] and generally poorer postoperative results when undergoing surgery [20,21].

Biofilm-forming bacteria have been proven to be extremely resistant to both the immune response of the host [22] and to antibacterial treatments due to their multidrug tolerance [23]. In an attempt to find a solution, a host of therapeutic strategies have been and are still being actively researched [24,25,26], but as of this point not a single one has emerged to provide an effective, safe, and long-lasting resolution.

BBF formation is typically described as a five-step process with surface attachment constituting the initial phase [26,27,28]. However, it is still unclear which factors contribute to the development of a BBF, although research has been conducted concerning several factors that include but are not limited to: air pollution, tobacco smoke, and prior surgery [29,30,31]. Another factor that may play a pivotal role in the attachment phase of BBF formation is the amount of mucin glycoproteins produced by the mucosa of the upper airways and the sinuses [32,33]. The rheological properties of the mucus layer covering the airways are mainly determined by the mucins [34]. They also serve a key protective function by adhering to bacteria and thus facilitate their mucociliary clearance [35]. Two types of mucins are produced in the airway mucosa—membrane-bound mucins (MUC1, 3A, 3B, 4, 11–13, 15–18, and 20) and secreted mucins (MUC2, 5AC, 5B, 6–10, and 19) [35,36,37]. Despite the important roles mucins play in the normal physiology of the airways, their abnormal production has been linked to pathology [37,38,39] (including chronic rhinosinusitis [40,41]). Among the different types of mucins, MUC5AC, predominately produced by the goblet cells [40], and MUC5B, mainly produced by the submucosal glandular cells [40], have been studied the most due to their presence in both healthy and diseased airways. Different expression patterns have been reported for MUC5AC and MUC5B between these two states, with diseased mucosa typically overproducing mucins [37,39,40].

The possible relationship between mucin overproduction and BBF formation has been studied both in vitro [42] and in vivo in patients undergoing surgery for CRS [32], and thus far a positive relationship has been suggested. Here, we evaluated this clinically significant relationship in 85 patient samples by means of both spinning disk confocal microscopy, which allowed us to determine the presence of BBF, and qRT-PCR, which we used to quantify the levels of expression of *MUC5AC* and *MUC5B*. We observed a significantly higher fraction of BBF-positive samples in the CRS group compared to control healthy individuals. In addition, higher expression of the *MUC5B* gene but not *MUC5AC* was discovered in the CRS group, thereby signaling a possible role for MUC5B overproduction in BBF development. Finally, we investigated the possible link between BBF presence/absence and mucin expression levels but did not find a significant correlation between them, which suggested that this relationship is not a straightforward one and that other patient-specific factors also contribute to chronic nasal cavity pathologies.

## 2. Materials and Methods

### 2.1. Study Design, Patients and Tissue Collection

For this prospective study, tissue samples were collected from 85 patients undergoing functional endoscopic sinus surgery (FESS) under general anesthesia in the Clinic of Otorhinolaryngology at the University Hospital “Tsaritsa Yoanna—ISUL”. The patients were treated for either chronic pathology of the nose and sinuses (CRS group, 71 patients) or acute nasal trauma (control group, 14 patients). All patients were above the age of 18, and the mean age was 44.92. Of all the patients, 57 were male (67.05%) and 28 (32.94%) were female. Information on previous nasal and sinus surgery, intranasal steroid therapy, and tobacco use was recorded, and all participants completed the SNOT-22 questionnaire [43] before the surgery took place. Two tissue samples were obtained from each patient—one for the evaluation of BBF status and another to determine the expression levels of the *MUC5AC* and *MUC5B* genes, with the mucosa being taken from the area of the uncinate process due to its key location within the ostiomeatal complex and its frequent use in other such studies [16,32,44,45,46,47]. Five patients (all from the CRS group) were excluded due to poor quality or insufficient quantity of the obtained samples. The study was approved by the Ethical Committee of Medical University—Sofia, and written informed consent was signed by every patient.

### 2.2. Bacterial Biofilm Identification

#### 2.2.1. Sample Staining and Image Acquisition

Following the FESS procedure, patient samples were immediately transferred to Dulbecco’s Modified Eagle Medium (DMEM, Thermo Fisher Scientific, Cat# 41966-029) in order to preserve cell viability until staining. The samples were stained with the LIVE/DEAD™ BacLight™ Bacterial Viability Kit (Thermo Fischer Scientific, Cat# L7007) according to the manufacturer’s instructions. Briefly, the samples were washed gently several times with phosphate-buffered saline (PBS, Merck, Cat# 524650) to remove blood and cell culture leftovers and then promptly stained for 20 min. The staining solution contained a combination of two organic dyes: Syto9, a green dye that stains live bacterial and eukaryotic cells; and propidium iodide (PI), a red dye that stains dead or dying cells (those with compromised cell membrane integrity). Therefore, this staining procedure allowed for an unbiased viability estimation of both the biofilm bacteria and nasal cavity epithelial cells.

Stained samples were mounted in 35 mm glass-bottom Petri dishes (MatTek, Cat# P35G-1.5-14-C) in such a manner that the epithelium pointed downward toward the glass bottom of the dish. Samples were overlaid with a cover glass so that they were gently pressed against the glass bottom, which was necessary for bubble eviction from under the sample to facilitate the subsequent microscope observations. Image acquisition was accomplished with an Andor Revolution spinning disk confocal system (Andor, Oxford Instruments). The glass-bottom Petri dishes were mounted on an inverted Nikon Ti-Eclipse microscope (Nikon) and were observed through a Nikon CFI Plan Apo VC 60x water immersion objective (Nikon) with a numerical aperture (NA) of 1.20. A 488 nm laser with a nominal power of 30 mW attenuated to 20% was used for Syto9 excitation, and a 561 nm laser with nominal power of 50 mW attenuated to 8.3% was used for PI imaging. Depending on the quality of the staining procedure, exposure times varied between 50 ms and 200 ms. Signal detection was accomplished via a high-sensitivity Andor iXon 897 electron multiplying charge-coupled device (EMCCD) camera (Andor, Oxford Instruments). Samples were meticulously inspected for both green and red fluorescence, and images from several representative regions were acquired for each sample. For every region, Z-stacks (>40 focal planes with 0.5 μm spacing) in both channels were obtained. Such an imaging procedure allowed us to obtain images through the entire depth of the epithelial layer and the mucus sheet above it, therefore greatly facilitating the assessment of epithelial health and biofilm presence and density.

#### 2.2.2. Biofilm Criteria

Using the BacLight dying protocol, bacterial biofilms were defined as areas of clustered fluorescence with elements of appropriate bacterial size (0.5–3 μm) and shape that were arranged in a characteristic three-dimensional structure. A less intense ‘‘blush’’ surrounding the areas of discrete, brightly fluorescing areas (i.e., the bacteria) was deemed to represent the exopolysaccharide matrix of the biofilm. Two blinded observers then analyzed the images and determined their BBF status.

### 2.3. MUC5AC and MUC5B Expression Level Quantification

All patient samples collected for gene expression analysis of *MUC5AC* and *MUC5B* were stored in RNAlater solutions (Thermo Fisher Scientific, Massachusetts, USA, Cat# AM7020) and frozen at −80 °C until further use. Total RNA was extracted from the tissue samples using the RNeasy Plus Micro Kit (Qiagen, Germany, Cat#/ID: 74034) according to the manufacturer’s instructions. The quantity and quality of the isolated RNA were quantified spectrophotometrically with NanoDrop (Thermo Fisher Scientific, United States). A total of 100 ng of RNA was used for the cDNA synthesis with a High-Capacity cDNA Reverse Transcription Kit (Thermo Fisher Scientific, United States, Cat# 4368814). The quantitative real-time reverse transcription PCR (qRT-PCR), which was accomplished by using the QuantiTect SYBR Green PCR Kit (Cat. No./ID: 204243) and QuantiTect Primer Assay (Qiagen, Germany, Cat#QT01322818, Cat# QT01329615), was performed on a 7900 HT Fast Real-Time PCR System (Applied Biosystems). The glyceraldehyde 3-phosphate dehydrogenase (*GAPDH*, Cat#QT00079247) gene was used as a reference control for normalization. Each reaction was performed in triplicate according to the manufacturer’s protocol. The relative quantification (RQ) of gene expression levels of *MUC5AC* and *MUC5B* was conducted using the 2^−ΔΔCt^ method.

### 2.4. Statistical Analysis

Statistical analyses were performed using SPSS 20.0 for Windows. Relationships between study groups were evaluated by using the chi-squared test. A two-tailed *p*-value < 0.05 was considered statistically significant.

## 3. Results

### 3.1. Patient Demographics

We collected a total of 170 tissue samples from the 85 patients that were recruited for this study—two samples from each patient. One of the samples was intended for evaluating the biofilm status and the other for quantifying *MUC5AC* and *MUC5B* expression levels. A total of 71 patients diagnosed with chronic rhinosinusitis were included in the CRS group, and the remaining 14 patients—individuals with no previous history or present diagnosis of chronic rhinosinusitis who were admitted for treatment of acute nasal trauma—served as controls (control group) as was done in other studies on the topic of CRS [40,48,49]. From the 71 patients in the CRS group, 5 were excluded due to poor quality or insufficient quantity of the obtained samples, and the remaining 66 comprised 44 male and 22 female patients. Their mean age was 47.24 years (range: 19–85); 48 out of the 66 had the clinical phenotype of CRS with nasal polyposis (CRSwNP) [50,51] (with 3 of them having undergone previous surgery for the same pathology); the remaining 18 did not have polyps (clinical phenotype of CRS without nasal polyps (CRSsNP) [50,51]), and there were no cases of previous surgery. Smoking was practiced by 26 people, and the remaining 40 were non-smokers. A total of 41 had tried therapy with nasal steroids prior to surgery with varying degrees of success but never with full or lasting relief of the symptoms. Only one patient had asthma, and 18 reported having an allergy. In terms of disease severity, the mean SNOT-22 score was 41.15. Of the 14 patients in the control group, 10 were male and 4 were female. Their mean age was 32.71 years (range, 18–74). All were admitted for surgical treatment due to acute nasal trauma and had no history of chronic inflammation in the nasal cavity or the sinuses. One had a history of prior surgery in the nasal region (reduction of a prior nasal fracture). Smoking was practiced by three people, and the remaining 11 were non-smokers. None had tried or were undergoing any kind of topical nasal treatment (including with steroids) before suffering the acute trauma. Three patients reported having an allergy, and none reported asthma. Their mean SNOT-22 score was 10.85. The demographic characteristics of the patients in both groups can be seen in Table 1 with more detailed information on each patient available in the supplementary materials (Table S1).

### 3.2. Evaluation of Bacterial Biofilm Status

**Figure 1:**
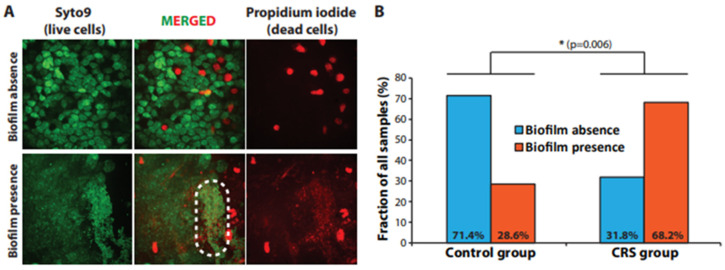
Evaluation of bacterial biofilm status. (**A**). Microscopic detection of BBF in patient samples. The dashed white ellipse encircles a dense BBF region. Presented images are maximum-intensity projections of the Z-stacks obtained experimentally. (**B**). BBF occurrence in the control group and the CRS group. Statistical significance was assessed using the chi-squared test.

In order to evaluate the patient tissue samples from both the CRS group and the control group for the presence of a bacterial biofilm, we dyed the samples with the LIVE/DEAD BacLight dye mix. Since Syto9 (the green dye in the mix) dyes only live cells and propidium iodide (PI, red dye) enters only dead cells with compromised cell membrane integrity, we managed to visualize both bacterial cells and eukaryotic epithelial cells and at the same time discriminate between live and dead cells (Figure 1A). By using this method, we accepted the disadvantage of not being able to identify the bacterial species that formed the biofilms; however, this also allowed us to pinpoint all biofilm-positive samples regardless of the species involved [10]. Image acquisition was accomplished via spinning disk confocal microscopy, which allowed for highly sensitive fluorescence detection and significant depth of investigation (Z-stacks through the entire epithelial and mucous layers were obtained). We discovered a bacterial biofilm structure in 45 out of the 66 patients in the CRS group (68.2%), while in the rest (21 samples or 31.8%) no such findings were made, so these samples were labeled as biofilm-negative (Figure 1B). Notably, only 4 out of 14 patients from the control group were identified as biofilm-positive (28.6%), while all other (10 samples, 71.4%) were negative for the presence of BBF (Figure 1B). With regard to the two types of CRS patients—those with and without nasal polyps—we discovered a BBF in 33 out of 48 (68.75%) patients with CRSwNP and in 12 out of 18 (66.67%) of the patients without polyps (CRSsNP).

The difference between the CRS group and the control group with respect to BBF status was highly statistically significant (*p* = 0.006), thereby showing a possible association between bacterial biofilm formation and chronic rhinosinusitis pathology in our patient cohort.

### 3.3. Evaluation of MUC5AC and MUC5B Expression Levels

**Figure 2:**
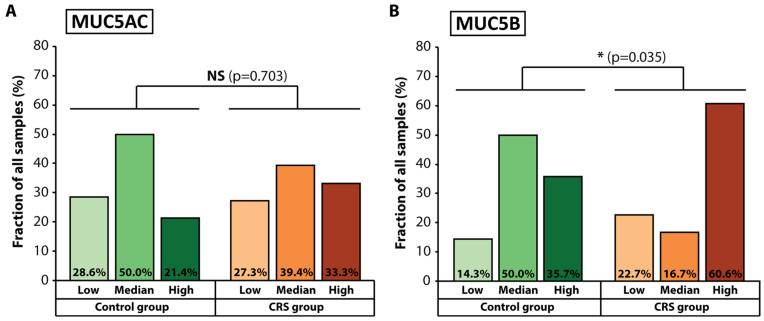
Evaluation of *MUC5AC* and *MUC5B* expression levels: (**A**). *MUC5AC* expression levels in the control group and the CRS group; (**B**). *MUC5B* expression levels in the control group and the CRS group. Low expression—RQ < 0.5; median expression—RQ between 0.5 and 2.0; high expression—RQ > 2.0. Statistical significance was assessed using the chi-squared test.

Next, we sought to determine the expression levels of the *MUC5AC* and *MUC5B* genes in all patient samples because the respective mucins secreted by nasal epithelial cells have been implicated in chronic rhinosinusitis pathology. To this end, we applied qRT-PCR to total RNA isolated from the patient samples. We used the expression levels of the housekeeping gene for glyceraldehyde 3-phosphate dehydrogenase (*GAPDH*) as a reference. The expression levels of *MUC5AC* and *MUC5B* were calculated as a relative quantity (RQ) value using the 2^−ΔΔCt^ method. In order to comprehensively analyze the expression profiles of the patient samples, we subdivided them in three groups according to their RQ values—low expression (RQ < 0.5), median expression (RQ values between 0.5 and 2.0), and high expression (RQ > 2.0). We discovered that 4 (28.6%) of the patients in the control group and 18 (27.3%) of the patients in the study group exhibited low levels of expression of *MUC5AC* (Figure 2A). Median levels of expression were observed in 7 (50%) control patients and 26 (39.4%) CRS patients. Interestingly, while only 3 patients (21.4%) from the control group exhibited high expression of *MUC5AC*, a total of 22 patients or 33.3% from the study group showed increased levels of *MUC5AC* expression. However, the differences between the two groups were not statistically significant (*p* = 0.703). Concerning *MUC5B* expression levels, we observed that 2 patients from the control group (14.3%) and 15 patients from the CRS group (22.7%) had decreased expression levels (Figure 2B). Notably, while 7 patients from the control group (50%) exhibited median levels of expression, only 11 patients (16.7%) from the CRS group did. The differences became even more prominent when comparing patients with high expression levels of *MUC5B*—5 patients from the control group (or 35.7%) displayed increased *MUC5B* expression versus 40 patients (or 60.6%) in the CRS group. Hence, we observed a significant difference between *MUC5B* expression levels in the CRS group compared to the control group (*p* = 0.035), thus prompting the notion that there was a possible link between high levels of *MUC5B* expression and the development of chronic inflammation in the area of the nasal cavity and the sinuses.

### 3.4. Investigating the Possible Relationship between Bacterial Biofilm Formation and Mucin Expression Levels

**Figure 3:**
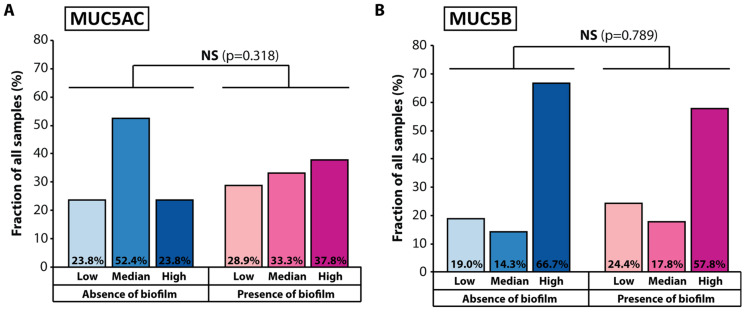
Relationship between bacterial biofilm formation and mucin expression levels among the patients in the CRS group for (**A**) *MUC5AC* and (**B**) *MUC5B*. Statistical significance was assessed using the chi-squared test.

A tempting hypothesis that was elaborated in several previous studies [32,33] is that there exists a link between mucin production by the nasal mucosa and the propensity for bacterial biofilm formation, hence chronic rhinosinusitis pathology. To reveal such a possible link in our patient cohort, we compared the levels of expression of *MUC5AC* and *MUC5B* with the presence or absence of bacterial biofilm for all the patients in the CRS group. We did not find a significant association between biofilm formation and the expression levels of both *MUC5AC* (*p* = 0.318) and *MUC5B* (*p* = 0.789) (Figure 3). However, it should be noted that 52.4% of the samples with no BBF detected displayed median expression levels of *MUC5AC* and 23.8% exhibited high expression levels, while in the biofilm-positive samples only 33.3% had median expression and 37.8% showed increased level of *MUC5AC* expression, thereby showing a possible marginal contribution of *MUC5AC* to BBF formation, which can be further explored in more extensive clinical studies.

## 4. Discussion

Chronic inflammations of the nasal cavity and sinuses (including chronic rhinosinusitis) are an intricate group of diseases with far-reaching consequences that are both social [4,52] and economic [5,53]. Multiple factors have been implicated in the development and aggravation of CRS such as exposure to tobacco smoke (either through active smoking or passive exposure [54]), allergies [55,56], bacterial superantigens [57], and environmental conditions [3]. A contribution of bacterial biofilm formation to the chronification of inflammations in the nasal cavity and sinuses is often proposed, and biofilm presence in diseased tissues has been confirmed multiple times [10,16,17,18]. In addition, dysregulation of mucin expression by nasal epithelial cells is also regularly cited as a possible cause for chronic nasal pathologies [40,41]. However, if and how these two factors are affiliated is largely unknown. Discovery of a connection between bacterial biofilm colonization of the mucosa of the upper airways and the expression profile of mucin genes—apart from the purely theoretical contribution—could have valuable practical implications. For example, abnormal expression of mucin genes could serve as a predictor in postoperative recovery and the probability of bacterial biofilm recolonization; hence, mucin expression levels could be included among the indications or contraindications for surgery. Expanding our knowledge of why certain people have a propensity to form bacterial biofilms and develop chronic infections in the mucosa of the nasal cavity and sinuses would be a vital step toward a better understanding of the subject and its eventual resolution.

Herein, we investigated the formation of bacterial biofilms and *MUC5AC* and *MUC5B* gene expression levels in samples from 80 patients admitted for either acute nasal trauma (14 patients, control group) or chronic nasal pathology (66 patients, CRS group). To this end, we used spinning disk confocal microscopy (SDCM) to evaluate the bacterial biofilm status of all samples and quantitative reverse transcription polymerase chain reaction (qRT-PCR) to quantify the *MUC5AC* and *MUC5B* expression levels. SDCM complemented with BacLight™ staining allowed us to not only detect the presence of bacterial biofilms but also to assess the viability of the bacterial cells and that of the underlying layer of nasal epithelial cells (Figure 1A), while qRT-PCR offered a fast and specific method to measure and compare *MUC5AC* and *MUC5B* expression across the patient samples. Importantly, our working pipeline may be easily reapplied in the clinical setting for biofilm status evaluation and mucin expression quantification whenever required.

Our results concerning bacterial biofilm formation uncovered a significantly larger fraction of BBF-positive samples in the CRS group compared to the control group (68.2% vs. 28.6%, respectively) (Figure 1B). Notably, the percentage of CRS patients discovered in our study to have a bacterial biofilm was in the range reported in several other publications, although that percentage varied widely depending on the study design and methods used (by between 25% and 92%) [10]. Our results suggested that there exists a positive relationship between bacterial biofilm formation and chronic nasal pathology and added to the body of evidence supporting the hypothesis that bacterial biofilms likely play an important role in the pathogenesis of CRS. Hence, routine BBF status evaluation in patient samples may prove to be an important clinical indicator for the comprehensive assessment of CRS pathology.

In a set of 20 articles examining the relationship between *MUC5AC* expression levels and CRS, 13 reported significantly elevated expression [32,40,41,44,45,58,59,60,61,62,63,64,65], 4 reported no significant difference in *MUC5AC* expression levels between CRS patients and control individuals [66,67,68,69], and 3 found decreased expression levels in CRS patients [37,70,71]. Regarding *MUC5B*, 9 out of 14 articles reported increased expression in CRS patients [32,37,40,41,44,65,67,72,73], 4 reported no significant difference [68,69,74,75], and a single article reported a decrease in *MUC5B* expression in the CRS group compared to the control group [70]. However, it must be noted that various study designs and different experimental methods were employed in these studies to uncover the link between mucin gene expression levels and chronic pathologies of the nasal cavity and sinuses [76]. In order to meticulously investigate the possible relationship between *MUC5AC* and *MUC5B* expression and CRS pathology in our patient cohort, we subdivided patient samples from both the control group and the CRS group into three subgroups—those with low, median, and high expression levels for both genes. Although we did not find a statistically significant difference between the control group and the CRS group concerning the expression of *MUC5AC*, it is important to note that we observed a higher percentage of patient samples with high expression levels and a lower percentage of samples with median expression of *MUC5AC* in the CRS group compared to the control group (Figure 2A). Increasing the patient cohort size in future studies could strengthen this observation for *MUC5AC*. Importantly, we discovered a considerably higher fraction of patient samples with increased expression and a lower fraction of samples with median expression levels of *MUC5B* in the CRS group compared to the control group (Figure 2B). These results may indicate a probable association between the expression of the Mucin 5B gene and chronic rhinosinusitis pathology in our patient cohort.

Finally, we probed whether there exists a connection between BBF status and mucin gene expression levels in our patient cohort. We did not find a significant relation between the presence of bacterial biofilms and *MUC5AC* and *MUC5B* expression levels in the CRS group, although we observed a higher percentage of *MUC5AC*-overexpressing patient samples among the BBF-positive part of the cohort (Figure 3). An interesting point of discussion was a comparison of the present study to another one that sought a relationship between bacterial biofilm formation and MUC5AC and MUC5B levels [32]. In contrast to the results of our study, which did not find a significant association between the presence of bacterial biofilms and mucin gene expression levels, Mao et al. reported that increased MUC5AC and MUC5B levels were associated with biofilm formation in CRS patients [32]. These distinct results may have been due to key differences between the two studies regarding the methods used for determining both bacterial biofilm status and the levels of mucin presence, the patient populations, the statistical methods employed to assess the patient data, and the multifactorial nature of CRS infections. We anticipate that future research will benefit from taking into account both studies when attempting to add more clarity to the topic.

In conclusion, as part of this study we performed bacterial biofilm status evaluation and *MUC5AC* and *MUC5B* gene expression quantification in both control and chronic rhinosinusitis patient samples in order to shed light on the possible link between these factors and CRS pathogenesis. We uncovered a solid relationship between BBF presence and CRS pathology in our patient cohort, thereby strengthening the notion that BBF formation is connected to the development of chronic nasal pathology. In addition, we reported that high expression levels of *MUC5B* (but not *MUC5AC*) could contribute to CRS because we observed a significantly higher fraction of *MUC5B*-overexpressing patient samples in the CRS group of the cohort. Finally, we did not discover a significant correlation between BBF status and mucin gene expression levels, which indicated that either these factors may independently contribute to CRS pathology or that their inter-relationship is not a straightforward one and is probably influenced by various other patient-specific factors.

## Figures and Tables

**Table 1:** Patient demographics.

	Mean Age (Years)	Sex (Men/Women)	Smoking	Nasal Polyposis	Therapy with Nasal Steroids *	Allergy	Mean SNOT-22 Score
CRS group	47.24	44/22	26/66 (39.39%)	48/66 (72.72%)	41/66 (62.12%)	18/66 (27.27%)	41.15
Control group	32.71	10/4	3/14 (21.42%)	none	none	3/14 (21.42%)	10.85

* Attempted therapy with nasal steroids prior to surgery.

## Data Availability

All data can be provided upon reasonable request addressed to the corresponding author.

## Supplementary Materials

The following supporting information can be downloaded at: https://www.mdpi.com/article/10.3390/jcm12051808/s1, Table S1: Patient demographics.
